# In memoriam Konstantin L'vovich Ivanov

**DOI:** 10.5194/mr-2-341-2021

**Published:** 2021-05-26

**Authors:** Alexandra Yurkovskaya, Geoffrey Bodenhausen

**Affiliations:** 1 International Tomography Center, Siberian Branch of the Russian Academy of Sciences, Novosibirsk, 630090, Russia; 2 Novosibirsk State University, Novosibirsk, 630090, Russia; 3 Laboratoire des Biomolécules, LBM, Département de chimie, École normale supérieure, PSL University, Sorbonne Université, CNRS, 75005 Paris, France

Our esteemed colleague and good friend Konstantin (“Kostya”) L'vovich Ivanov has become one of the first victims of the pandemic in our community. He passed away in a hospital in Novosibirsk on 5 March 2021. We shall deeply miss Kostya as an exceptional human being: he was a creative yet rigorous scientist, a generous and attentive friend, and a considerate and eminently civilised colleague. Rob Kaptein wrote that “[Kostya] was not only a great scientist, one of the few people who really understood magnetic resonance and spin chemistry, but also a good human being, always sincere, honest, and considerate. In addition, he was a great citizen of the scientific community; aside from his demanding job as the director of the International Tomography Center (ITC) of the Siberian Branch of the Russian Academy of Sciences, he kept his research at a high level and organized a multitude of meetings, seminars and webinars.” Kostya had built a strong relationship with Rob when they worked together in Novosibirsk, with the support of a Russian “megagrant” that Kostya had helped to secure. Kostya started further initiatives, so that another similar grant may soon be awarded where one of the undersigned may come to play a role, alas without Kostya's diplomatic and scientific skills.

Rolf Boelens wrote that “[Kostya's] theoretical knowledge on NMR was phenomenal, discussing [with him was] very stimulating and never dull. [...] It was clear that Kostya was not only an outstanding scientist, but also an excellent teacher, who very well trained and stimulated his students.” Indeed, Kostya was a master of many trades, hyperpolarization, chemically induced dynamic nuclear polarization, diffusion-controlled reactions, parahydrogen-induced polarization using catalysts in both low and high fields, field-dependent relaxation, long-lived states, and magnetic resonance imaging, and he nurtured some challenging aspects of quantum mechanics such as avoided level crossings with great talent.


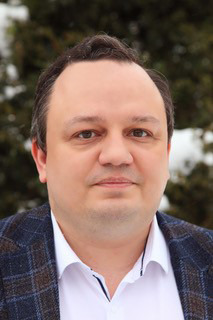


One of his colleagues in Novosibirsk wrote that “Kostya could be a humble and cheerful easy-going person, and yet be a leader with a steel hand realizing his vision of how things should be done despite numerous obstacles, never afraid of stepping up front and standing for his words. He was a lucky guy, and he passed his luck over to people whom he met. Looking back now, it is incredible how much he had accomplished in his short but fruitful life, and how much he had started that we can continue on. It is our obligation to him to keep things that he initiated going and developing, this will be the best possible homage to Kostya.”

Kostya Ivanov studied at the Faculty of Physics of Novosibirsk State University, obtained the degrees of Bachelor of Science (1998), Master of Science (2000), and PhD (2002) under the supervision of Renad Sagdeev and Nikita Lukzen at the International Tomography Center (ITC), Novosibirsk, and Doctor of Science in Physics and Mathematics (2008), and was appointed Professor of the Russian Academy of Sciences.

Kostya held a Fellowship of the Alexander von Humboldt Foundation (Germany) in 2008, where he worked with Hans-Martin Vieth, and a Fellowship of the Japanese Society for the Promotion of Science in 2016 that allowed him to work with Takeji Takui and Kazunobu Sato. He received the Medal of the European Academy of Science (Europea Academia Prize) in 2010 and shared the Laukien Prize with Simon Duckett and Warren Warren in 2020.

Kostya recently spent a month in Paris as professeur invité at ENS. As Fabien Ferrage wrote, “Kostya had so much strength, he was so intelligent, knowledgeable, hard-working and generous. We were of the same age, and his daughter Kseniya and my son Ravi also are of nearly the same age, which gives an even greater sense of proximity to this tragedy.”

Kostya was a key member of the “Division of hyperpolarization” of the Groupement Ampere. Kostya was also one of the driving forces behind the creation of *Magnetic Resonance*, the open-access journal of the Groupement Ampere. Only a few months ago, Kostya, Joerg Matysik, Rolf Boelens, Daniel Abergel, and Fabien Ferrage had taken the initiative to compile two special issues of *Magnetic Resonance* on the occasion of the 80th birthday of Rob Kaptein and the 70th of one of the undersigned. Our community will bitterly miss Kostya when these special issues will be presented at events that Kostya would surely have wished to attend.

We shall also miss Kostya as one of the leading figures of the “Intercontinental NMR Seminar Series” (ICONS, now appropriately named after him), both in the form of regular “zoominars” and in the form of virtual meetings extending over 3 consecutive days. It is clear to all of us that Kostya gave a vital impetus to such meetings, which are not merely an adequate response to the current pandemic that would be fatal to him, but which may also prefigure the future of many international meetings, where we shall bitterly miss Kostya's participation as our colleague and friend.

We shall remember Kostya, not only for his remarkable achievements in many areas, but also for his energy, his ability to make things happen, his generosity, and his friendship. Please join us in extending a message of condolence to his wife Elena, his daughter Kseniya, and his colleagues at the ITC in Novosibirsk that he directed with unique skills.

